# Supplementary material for: Complexome profiling of the Chlamydomonas psb28 mutant reveals TEF5 as an early PSII assembly factor
Source: Plant Cell. 2025 Mar 17;37(6):koaf055. doi: 10.1093/plcell/koaf055 (PMC12164590; doi:10.1093/plcell/koaf055)
Supplement: koaf055_Supplementary_Data [file koaf055_supplementary_data.zip › psb28_interactive.zip!/plots/intensity/qualityPlots/peptides/_Cre17.g703450.t1.1.html]

### Cre17.g703450.t1.1

Trivial name:   
SeqCoverage [median]: 1.40  
Mass: 172.94 kDa  
  
 

| Sequence | MissedCleavage | Mass [Da] | wtIntensity | muIntensity | Score |
| --- | --- | --- | --- | --- | --- |
| VITVMTAESVDVRDR | 1 | 1689.87 | 155.72 | 0.00 | 37.95 |
| VQLEGVYLR | 0 | 1075.60 | 916.59 | 0.00 | 65.04 |
|  |  |  | 1072.3 | 0.0 |  |
